# Effect of community-based health insurance on catastrophic health expenditure among chronic disease patients in Asella referral hospital, Southeast Ethiopia: a comparative cross-sectional study

**DOI:** 10.1186/s12913-023-09181-5

**Published:** 2023-02-23

**Authors:** Mosisa Bekele Degefa, Berhan Tassew Woldehanna, Anagaw Derseh Mebratie

**Affiliations:** 1 Department of Public Health, College of Health Science, Arsi University, Asella, Ethiopia; 2 School of Public Health, Addis Ababa University, Addis Ababa, Ethiopia

**Keywords:** Chronic disease, Catastrophic health expenditure, Community-based health insurance, Ethiopia

## Abstract

**Background:**

Chronic disease-related catastrophic health spending is frequent in Ethiopia affecting several households, particularly the poorest ones. A community-based health insurance (CBHI) scheme has been in place in Ethiopia since 2011. The scheme aims to provide financial protection against health expenditure but there is little evidence of how well it protects chronic patients financially.

**Objective:**

The objective of the study was to evaluate the effect of community-based health insurance in reducing the incidence of catastrophic health expenditure among patients attending chronic disease follow-up departments in Asella referral hospital, Southeast Ethiopia.

**Method:**

A health facility-based comparative cross-sectional study was conducted in Asella referral hospital from March 2022 to May 2022. Systematic random sampling was used to select 325 chronic patients. Data were collected using an open data kit (ODK) collect app and then imported to STATA version 16 for analysis. Propensity score matching was used to evaluate the effect of community-based health insurance on catastrophic health expenditure.

**Result:**

The study enrolled a total of 325 chronic patients (157 insurance members and 168 nonmembers). More than 30% of the study participants incurred health spending that could be catastrophic based on the 15% nonfood threshold. Catastrophic health expenditure was found in 31% of insured and 47% of uninsured participants. Overshoot and mean positive overshoot were 10% and 33% for insured members, respectively and the corresponding figures were 18% and 39% for nonmembers. Community-based health insurance contributes to a 19% ((ATT = -0.19, t = -2.97)) reduction in the incidence of catastrophic health expenditure among chronic patients. This result is found to be consistent for alternative measurements of the outcome variable and the use of alternative matching algorithms.

**Conclusion:**

Chronic patients, particularly those in uninsured households, had a high incidence and intensity of catastrophic health expenditure. Hence, it is relevant to expand community-based health insurance to provide financial protection for people suffering from chronic conditions.

## Background

Chronic diseases such as diabetes mellitus, cardiovascular disease and cancer, have become major problems around the world [1, 2]. The rising costs of these diseases have made it increasingly difficult for patients to afford medical costs since such diseases require frequent interactions with the health system. Particularly when health systems are mainly financed through out-of-pocket payments(OOP), they can cause impoverishment [2].

Catastrophic health expenditure (CHE) that surpasses a certain threshold threatens a household's financial ability to meet its basic necessities of families in financial distress [3, 4]. CHE due to chronic diseases is occurs in 6–84% of households depending on the chosen catastrophe threshold [3–5]. The financial burden of noncommunicable diseases (NCDs) is exacerbated in low- and middle-income countries (LMICs), where 6–11% of the population would be impoverished (at 1.25 USD/day poverty line) if they had to buy the cheapest generic diabetic medicine [5]. OOP payments in Ethiopia was estimated to be 18.2 billion ETB in 2015/16, with noncommunicable illness treatment accounting for 23% of overall OOP spending [6–8]. Various studies in Ethiopia also indicated that CHE affects between 27% and 88.4% of households [9–12].

A sustainable way to reduce reliance on OOP in low and middle-income countries is argued to be developing prepayment arrangements [13]. In this regard, community-based health insurance (CBHI) schemes are considered to be one alternative to overcome CHE and achieve universal health coverage [14–16].

CBHI has been endorsed in Ethiopia since 2011 as part of the health financing reforms that target strengthening domestic resource mobilization and providing financial protection against OOP health spending. Initially, the scheme was introduced in 13 pilot districts, and then the scheme was expanded to 911 districts. The uptake of the scheme reached 58% of the target households and it extended coverage to approximately 8.6 million households or 40 million people [17].

The scheme premium is set at the household level and varies by region [18, 19]. The benefit package included both outpatient and inpatient services provided by public facilities. It does not cover cosmetic procedures and services provided for free [17, 18].

There is mixed evidence regarding the role of CBHI in reducing CHE in practice. According to studies conducted in India and Lao, it aids in lowering the CHE [20–22]. On the other hand, other studies found that health insurance is ineffective in preventing CHE in families with chronic disease patients in China [36, 37]. However, despite the high burden of catastrophic health expenditure, little is known about the effect of CBHI in mitigating CHE among chronic disease patients [12, 24]. Hence, this study aims to assess the effect of community-based health insurance in providing financial protection for individuals with chronic diseases.

## Methodology

### Study setting and design

A health facility-based comparative cross-sectional study was used to collect data from the source population. Data were collected from Asella Teaching and Referral Hospital, which provides medical services for more than 3.5 million inhabitants and 544,966 CBHI-enrolled households in the Arsi Zone. The hospital has been serving as a teaching hospital of the college of Health science and the college has specialty programs in internal medicine, surgery, pediatrics and gynecology, and obstetrics. It also has undergraduate teaching programs in medicine and public health. The study was conducted from March 15, 2022, to May 13, 2022 among chronic disease patients attending follow-up appointments at Asella Teaching and Referral Hospital.

### Population

The source population consisted of all patients with chronic diseases who attended follow-up appointments and the study population included those patients who were receiving care at Asella Teaching and Referral Hospital during the data collection periods.

### Inclusion and exclusion criteria

Patients who had been attending follow-up appointments for at least a month and agreed to participate in the study were included. However, individuals who were seriously ill and unable to respond as well as those who were under the age of 18, were excluded from the study. The study also excluded pregnant patients since most of gestational hypertension and diabetes are transient and not considered as chronic conditions.

### Sampling

The sample size was determined using both double and single-population proportion formulas. The proportion of CHE was taken from the final report of the CBHI evaluation, and it was 7% and 19% among insured and noninsured households, respectively [17]. 80% power, 95% CI, and 5% degree of precision with a one-to-one ratio among insured and noninsured were taken. Based on this formula, the calculated sample size was 278. For a single population proportion formula, the magnitude of CHE among diabetic patients was taken from a study conducted in Bahir Dar (59.6%) [9]. After adding a 10% nonresponse rate and making adjustments using a population correction formula, 336 chronic patients were recruited in the study, including 168 CBHI members and 168 nonmembers. We prefer to use the estimate based on single-population proportion formula as this yields a higher sample size and hence, a better power of the significance test, as compared to the double population proportion formula.

### Sampling techniques and procedure

The sample size was proportionally allocated to chronic disease follow-up OPD (diabetes, psychiatry, and other chronic diseases) in the hospital based on patient flow reports that amount to 800, 335, and 600 patients, respectively [25]. Consequently, proportionally allocated sample sizes were 155 for diabetes OPD, 65 for psychiatry and 116 for other chronic diseases. Then to obtain representative study participants, systematic random sampling was applied to every 5^th^ patient among eligible patients.

### Data collection and management

Data were collected using a structured questionnaire adapted from similar studies [9, 10, 17, 26]. The tool was first prepared using the English language and later translated into the local languages (*Amharic* and *Afan-Oromo*) since these languages are most widely spoken in the study area. Then it was back-translated into English to ensure the validity of the question. Pretesting was performed on 5% of the sample (17 chronic patients) and the necessary modifications were made based on the findings.

In line with the objective of the study, data were collected from chronic patients who attended follow-up appointments between March and May 2022 through a face-to-face interviews. To ensure the privacy of the respondents, data collection took place by setting up separate rooms for interviews. Responses of the study participants were automatically entered into the ODK collect app that was installed on tablets.

Under the careful supervision of the main investigators, one BSc nurse and one midwife who are not employees of Asella referral and teaching hospital gathered information from respondents. The data collector had two days of training on the objectives and data collection procedures to ensure the quality of the data.

### Measuring catastrophic health expenditure

Catastrophic health expenditure (${CHE}_{i}$) was measured as a binary variable using the following formula.

1where ${OOPh}_{i}$ refers to out of pocket health expenditure in the household, ${X}_{i}$ denotes household nonfood expenditure or total expenditure, and $z$ is a given catastrophic threshold.

${OOPh}_{i}$ captures both direct medical and direct nonmedical costs paid by the patients to be able to access medical services. Direct medical cost involve costs for drugs, consultations (registration fees) and laboratory costs incurred by the client during a one month follow up period. Direct nonmedical cost includes costs for transportation, food, and accommodation while seeking medical care in one month follow up period for both clients and caregivers.

To measure CHE, we used the approach proposed by Wagstaff and Van Doorslaer, (2002).

### Wagstaff and van Doorslaer methodology

The incidence and intensity of catastrophic expenditure were calculated using headcount, overshoot, and mean positive overshoot by using formulas 2 to 4 respectively. Catastrophic payment headcount is the percentage of households incurring catastrophic expenditure and it measures the magnitude of catastrophic health expenditure within the overall sample by using the following formula 2. 

2 $$H = \frac{1}{N}\sum_{ i=1}^{ N} {CHE}_{i}$$

The difficulty with catastrophic payment head count is it does not reflect the amount by which households exceed the threshold. Therefore, we measured catastrophic expenditure overshoot which captures the average degree to which health expenditure exceed the given threshold Z by using the following formula 3.

3 $$O= \frac{1}{N}\sum_{i=1}^{N}{CHE}_{i} \left[\frac{{T}_{i}}{{TE}_{i}-{TFE}_{i}}-Z\right] = \frac{1}{N}\sum_{i=1}^{N}{o}_{i}$$

The incidence and intensity of catastrophic expenditures are related through the mean positive overshoot (MPO) which captures the intensity of catastrophic expenditures defined as overshoot divided by headcount. MPO indicates the average percentage of OOP medical expenditure in excess of the threshold among households incurring CHE. It will be calculated using formula 4 below:

4 $$MPO= \frac{O}{H}$$

### Catastrophic threshold level

There is no universal consensus on catastrophic threshold level and different studies use different thresholds that range from 10% to 40% of total household expenditure, nonfood expenditure, and capacity to pay [17, 24, 26, 27]. In this study different thresholds were used to check for sensitivity of incidence and intensity of catastrophic expenditure. The thresholds include 15%, 25%, and 40% of nonfood expenditure as well as 10% and 15% of total expenditure. To evaluate the effect of CBHI on CHE 15% of nonfood expenditure was used since it was the most widely accepted country-specific threshold which is adapted from a national CBHI evaluation study [17, 24].

### Data processing and analysis

Data were collected using the ODK collect app. After completion of the data collection step, the data were checked and transferred to STATA version 16 for analysis.

Descriptive statistics were used to summarize the data and explain the study variables. Principal component analysis was used to compute the wealth index of the households. The index was created by aggregating a large number of household productive assets, nonproductive assets, and household amenities. All asset data were recoded as binary except for tropical livestock units (TLUs) and land size, which were used as continuous variables. Propensity score matching was used to estimate the effect of community-based health insurance on catastrophic health expenditure. This method helps to identify counterfactual outcomes for the interventions using a comparable group of households that do not participate in CBHI but had the same probability of participating based on a set of observable factors. PSM is also appropriate for our case since we do not have data generated through randomization procedure and there is no baseline study that shows outcomes for the study participants prior to launching the CBHI [22, 24, 28].

The effect of CBHI on the outcomes was estimated using multistep procedure. The first step involved fitting a logit model to compute propensity score using CBHI membership as the dependent variable and observable variables that affect enrollment in the CBHI program as covariates. These include age, marital status, educational status, household size, occupation, and wealth index.

In the second stage, the best matching algorithms were chosen using different criteria. The preferred algorithm was chosen based on having a higher number of matched samples, a low pseudo-R^2^ value, and a reduced mean difference between the treated and control groups. This criterion was used to test local linear matching, kernel matching with different bandwidths, radius matching with caliper, and nearest neighbor matching with and without replacement. The model interpretation is then based on nearest neighbor matching (*N* = 5), which is found to be a more efficient option.

The third stage involved evaluating PSM's validity, which depends on two crucial assumptions: common support and conditional independence. The common support assumptions were verified visually using histograms or density-distribution plots of propensity scores. The conditional independence assumption was verified using well-known balance tests, such as standardized bias, the t test, and pseudo-R^2^.

Finally, the average treatment effect on treated (ATT) was computed by averaging the difference between the CHE of the insured households and that of the noninsured households after matching using propensity scores.

### Operational definition

Chronic disease was defined as patients with noncommunicable diseases (mainly DM, cardiovascular disease, mental illness, and respiratory diseases) who had been on follow-up appointments for at least a month.

Community based health insurance members were households that had been members for at least a month and renewed their membership for the fiscal year of 2021/2022.

## Result

### Sociodemographic characteristics

In this study, 325 study participants were involved, with a 96.7% response rate. The sociodemographic characteristics of the respondents are reported in Table 1. Of these, 167 (51.38%) respondents were males. The age range of the respondents was 18 to 79, with a mean of 47.32 (SD + 16.47 (50)) years. The majority of study participants were married, Orthodox Christians and urban residents. The average household size of respondents was 4.73 (SD + 2.03(5)). In terms of educational attainment, 75 (23%) can read and write while 71 (22%) had completed tertiary education (above 12). Farmers accounted for 25% of the research participants followed by employees at 19%.

**Table 1:** Sociodemographic characteristics of respondents attending chronic follow up in Asella referral hospital, 2022 (n = 325)

*Characteristics*	*Category*	*Frequency*	*Percent*
*Sex*	Male	167	51.38
	Female	158	48.62
*Age in year*	18–30	70	21.54
	31–45	81	24.92
	46–60	106	32.62
	61–79	68	20.92
*Religion*	Orthodox	186	57.23
	Muslim	102	31.38
	Catholic	5	1.54
	Protestant	32	9.85
*Marital status*	Single	65	20
	Married	235	72.31
	Widowed	19	5.85
	Separated	6	1.85
*Residence*	Urban	214	65.85
	Rural	111	34.15
*Household size*	< = 5	220	67.69
	> 5	105	32.31
*Educational Status*	Not able to read and write	59	18.15
	Read and write	75	23.08
	Primary education (1–8)	57	17.54
	Secondary education (9–12)	63	19.69
	Tertiary education (above 12)	71	21.85
*Occupation*	Farmer	80	24.62
	Employee (Gov’t/private/NGO	64	19.38
	Self employed	2	0.62
	Unemployed	22	6.77
	Merchant	35	10.77
	Daily laborer	1	0.31
	House wife	50	15.38
	Student	25	7.69
	Retired	46	14.15
*Wealth quintiles*	Poorest	70	21.54
	Q2	61	18.77
	Q3	64	19.69
	Q4	65	20
	Richest	65	20

### Clinical characteristics

Table 2 presents the clinical characteristics of the study participants. Approximetly 143 (44%) were diagnosed with diabetes and 98 (30.15%) were hypertensive. The mean duration of living with chronic diseases for the study participants was 3.9 (SD ± 4.22) years. The majority (98.77%) of patients had regular follow up and among these patients 169 (52.65%) had follow up every month.

**Table 2:** Clinical characteristics of respondents attending chronic follow up in Asella referral hospital, 2022

*Characteristics*	Category	Frequency	Percent
*Type of chronic disease diagnosed*	Diabetes	143	44
	Hypertension	98	30.15
	Heart disease	60	18.46
	Mental illness	66	20.31
	Kidney disease	16	4.92
	Other^1^	8	2.46
	Total	391	120.31
Regular follow up	Yes	321	98.77
	No	4	1.23
*Follow up appointment*	Every month	169	52.65
	Every two months	102	31.78
	Every three months	47	14.64
	Other^2^	3	0.93

Other^1^:- Stroke and COPD Other^2^;- Every two week and Six months

### Catastrophic health expenditure

The study shows that more than one third of the study participants (30%) had encountered catastrophic health expenditure at 15% nonfood threshold. The incidence of catastrophic spending was higher for noninsured patients for almost thresholds and when different methods were employed. The incidence of catastrophic health spending among CBHI members was 31% compared to 47% for nonmembers. At 15% of total household expenditure, the incidences of CHE for insured and uninsured patients were 20% and 18%, respectively (Table 3).

**Table 3:** Incidence and intensity of CHE based on CBHI status among chronic disease patients attending follow up in Asella referral hospital based on CBHI status, 2022

OOP as share	CBHI enrollment Yes				CBHI enrollment No			
	10%	15%	25%	40%	10%	15%	25%	40%
**Nonfood**								
**Head count (%)**	44	30.6	21	10.2	63	47	27.7	13.9
**Overshoot (%)**	12	10.20	7.57	5.20	21	18.30	14.80	12.15
**MPO (%)**	27.23	33.22	36.02	50.83	33.87	39.39	54.06	88.74
**Total expenditure**								
**Head count (%)**	23	20			33.3	18		
**Overshoot (%)**	4.34	3.32			6.10	4.84		
**MPO (%)**	18.91	16.80			18.23	27.10		

The incidence and intensity of CHE were measured based on the Wagstaff and Van Doorslaer approach. The threshold for CHE utilized in total expenditure is less than 15% since the denominator is larger than other measures such as non-food expenditure

When comparing noninsured households to their insured counterparts, the intensity of catastrophic out-of-pocket health costs owing to chronic illness is high. At a 15 percent threshold, for example, the overshoot was 10% for members and 18% for nonmembers. In addition, the mean positive overshoot for insured and uninsured households was 33% and 39%, respectively.

### Effect of participating in CBHI on CHE

The effect of community-based health insurance on catastrophic health expenditure has been estimated using the average treatment effect on treated. This was estimated at the most widely acceptable threshold, which is 15% of nonfood expenditure. A total of 155 CBHI members and 166 nonmembers with chronic diseases were compared based on the nearest neighbor matching algorithm. Table 4 shows enrollment in CBHI had a significant negative effect on catastrophic health expenditure. For instance, at a 15% threshold level, patients who are members of CBHI had 19% lower incidence of catastrophic health expenditure compared to noninsured households. These results are found to be consistent for alternative dentition of CHE and for using alterative matching algorithms.

**Table 4:** Effect of community-based health insurance on catastrophic health expenditure among chronic disease patients on follow up in Asella referral hospital, 2022

Outcome Variable	Treated	Controls	Difference	S.E	T-stat
CHE at;					
10% nonfood	.438709677	.651612903	-.212903226	.064271973	-3.31^a^
15% nonfood	.303225806	.494193548	-.190967742	.064271037	-2.97^a^
40% nonfood	.096774194	.219354839	-.122580645	.044248702	-2.77 ^a^
10% Total expenditure	.225806452	.372903226	-.147096774	.060168301	-2.44 ^a^

^a^Significant at 1%

## Discussion

This study aimed to evaluate the effect of community-based health insurance on catastrophic health expenditure among patients attending chronic disease follow-up in Asella Referral Hospital, Southeast Ethiopia. It was found that, at the 15% nonfood threshold level, more than 30% of study participants faced catastrophic expenditures. CBHI members had a lower incidence of catastrophic health spending than non-CBHI members, with 31 percent for CBHI members and 47 percent for nonmembers. This is higher than the earlier CBHI evaluation study, which found that the incidence was only 7% and 19%, respectively [17]. The previous study focuses on health expenditure in general, while the current study focused on chronic conditions, which require frequent visits to health facilities and additional costs. The existing report also supports that the prevalence of CHE is greater among families with chronic disease patients [29, 30].

Moreover, catastrophic health payments are also found to be more severe for uninsured people than for insured people. For CBHI members, the catastrophic overshoot and MPO were 10% and 33%, respectively, but for nonmembers, they were 18% and 39%. The MPO showed that CBHI members and nonmembers with catastrophic health expenses invested 48% and 54% of their monthly non-food expenditure on chronic disease treatment, respectively. This suggests that individuals with chronic diseases are at risk of spending a considerable amount of income on chronic illness care.

The propensity score matching model found that CBHI membership lowers the incidence of catastrophic health spending by 19 percent. This is because the CBHI scheme benefit package included medical expenses, particularly drug and diagnostic costs, which make up the majority of out-of-pocket health expenditures for the treatment of chronic diseases. As the Ethiopian CBHI does not provide coverage for nonmedical costs, the reduction in health expenditure for insured patients is because of a reduction in direct medical costs. This finding is similar to a study conducted in Northeast Ethiopia, which found that insured patients have lower catastrophic out-of-pocket expenses by 23%. Furthermore, several studies in Ghana, Nigeria, India, and China reported that health insurance schemes reduce the incidence of catastrophic health expenditure as well as out-of-pocket payments for health services [20–22, 30–32].

In contrast, according to a study conducted in China, health insurance is ineffective in reducing the financial burden among chronic disease patients [33, 34]. This disparity could be attributable to differences in health insurance benefit packages. In China, the insurance benefit package covers inpatient and limited outpatient care, whereas, in Ethiopia, the CBHI benefit package involves both inpatient and outpatient services [17, 35].

### Limitations of the study

This study attempted to add knowledge to the literature by examining the effect of CBHI in reducing health speeding for highly vulnerable groups. However, it has the following limitations. The sample used in this study can only represent a portion of outpatient health spending; it is possible that they used inpatient health services as well. Sample size was also determined using single population proportion formula without considering potential differences in the incidence of CHE between insured and noninsured patients. Furthermore, the propensity score model used in this study ignores the effects of unobserved factors that could affect the study's outcomes. There could also be recall bias in the measurement of health expenditure as the study participants may not remember all their health spending. Hence, these issues need to be taken into account when interpreting the results of this study.

## Conclusion

The overall evidence in this study implies the catastrophic nature of chronic health conditions on the welfare of households. Chronic patients, particularly those in uninsured households, had a high incidence and intensity of catastrophic health expenditure. Hence, it is important to focus on addressing the problem by designing an alternative resource mobilization strategy to meet medical spending for chronic illness. In this regard, Community based health insurance had significant effect on reduction of the incidence of catastrophic health expenditure. Expanding the coverage of community-based health insurance schemes is relevant to provide financial protection for people suffering from chronic conditions, particularly for the poorest households.

## Data Availability

All the data used in this study are kept confidential and will be available from the corresponding author upon reasonable request.

## Abbreviations

CBHI: Community-based health insurance
CHE: Catastrophic health expenditure
CI: Confidence Interval
MPO: Mean positive overshoot
ODK: Open data kit
OOP: Out of pocket
PCA: Principal component analysis
SD: Standard deviation
